# Dissecting Virus Entry: Replication-Independent Analysis of Virus Binding, Internalization, and Penetration Using Minimal Complementation of β-Galactosidase

**DOI:** 10.1371/journal.pone.0101762

**Published:** 2014-07-15

**Authors:** Christine Burkard, Louis-Marie Bloyet, Oliver Wicht, Frank J. van Kuppeveld, Peter J. M. Rottier, Cornelis A. M. de Haan, Berend Jan Bosch

**Affiliations:** Virology Division, Department of Infectious Diseases and Immunology, Faculty of Veterinary Medicine, Utrecht University, Utrecht, The Netherlands; German Primate Center, Germany

## Abstract

Studies of viral entry into host cells often rely on the detection of post-entry parameters, such as viral replication or the expression of a reporter gene, rather than on measuring entry *per se*. The lack of assays to easily detect the different steps of entry severely hampers the analysis of this key process in virus infection. Here we describe novel, highly adaptable viral entry assays making use of minimal complementation of the *E. coli* β-galactosidase in mammalian cells. Enzyme activity is reconstituted when a small intravirion peptide (α-peptide) is complementing the inactive mutant form ΔM15 of β-galactosidase. The method allows to dissect and to independently detect binding, internalization, and fusion of viruses during host cell entry. Here we use it to confirm and extend current knowledge on the entry process of two enveloped viruses: vesicular stomatitis virus (VSV) and murine hepatitis coronavirus (MHV).

## Introduction

Viral infections pose one of the major public health threats of our time, as demonstrated by the emergence of the SARS-coronavirus (SARS-CoV) in 2002/2003 and the new pandemic influenza H1N1 virus in 2009. Viruses are obligatory intracellular pathogens, which depend on host cells for their replication. Understanding the viral life cycle and studying the cellular factors involved in viral infection are crucial for the identification of new antiviral targets and the development of antiviral drugs. As virus entry is the first step in the viral life cycle, inhibition of this essential process is an attractive approach to block virus infection [1]. Current methods for studying viral entry into host cells mostly rely on post-entry parameters, such as replication or the expression of a reporter gene, rather than on measuring entry *per se* [2]–[4]. Studying virus entry directly, i.e. in a virus replication-independent manner, has proven to be difficult, certainly when using low, physiologically relevant amounts of virus particles.

To study distinct virus entry stages (binding, internalization, penetration/fusion) a variety of methods have been applied. Radioactive labeling of structural viral components and electron microscopy (EM) of infected cells have been used to investigate virus binding and internalization [5]–[9]. Radioactive labeling of structural viral components, mostly using [^35^S]methionine-labeling, can be used mainly to observe binding, internalization, and low-pH induced membrane fusion [5], [10], [11]. In addition to requiring the handling of radioactive components and elaborate protocols, this technique does not allow observing virus fusion directly. The study of virus infections by EM has been used to study infections *per se* and the viral entry or release process (reviewed in [9], as well as [5], [8], [10], [11]). Even though EM techniques are able to give visual insight into virus entry, including various stages of the entry process, it is still difficult to identify cellular factors and pathways involved in the uptake process with this technique. Also, EM is very labor intensive, usually requires high virus concentrations, and is hardly suitably for medium or high throughput experiments. Virus entry has also been studied by fluorescence microscopy (FM), either by detecting replication-dependent viral protein or reporter-fusion protein expression or by imaging of fluorescently labeled virions. Investigating virus entry by FM of fluorescent reporter protein expression as the name already indicates requires viral replication. This process occurs long after viral entry and fusion has occurred and thus does not allow differentiating between entry and replication (e.g. [12]). The only way to partially differentiate the processes is to add perturbing agents in timely intervals. Investigating entry using fluorescently labeled virions by expression of structural fusion proteins or chemical labeling allows to investigate virus entry in further details, e.g. using co-localization, live-cell microscopy, or tracking studies (e.g. [10], [13]–[16]). Whereas FM reporter protein expression experiments may be used for high-throughput experiments and can be used for a wide variety of viruses, the study of fluorescently labeled virions is laborious, requires high magnification and resolution, and is rarely suited for non-enveloped viruses.

More specialized fusion assays have been developed over the last few decades. Early examples involved labeling of virions using self-quenching dyes or the activation of photosensitized labeling on virions by fluorescent lipids on target membranes [17]–[19]. However, these assays solely allow for the investigation of fusion and not other entry steps, and are very complex and difficult to adapt to non-enveloped viruses. Recently, enzymes have been employed as reporters for virus entry by incorporating them into virions to allow for investigation of entry independent of replication. Therefore either firefly- or gaussia luciferase, or β-lactamase have been incorporated as structural (lumenal) fusion proteins into virions [20]–[25]. However, the integration of an entire enzyme of several hundred amino acid in size can severely affect virus assembly and/or infectivity. Also only fusion towards the cytosol may be investigated in intact cells. When using the assays by lysing cells it cannot distinguish between internalized and fused virions. The enzymatic assays published so far, with the exception of gaussia-tagged vaccinia virus [25], have been mainly used for fusion measurements only. While all of the above-mentioned methods have their strengths and weaknesses and have proven useful, the lack of assays that distinctly detect the different steps in viral entry hampers the analysis of this important process significantly. There is a clear need for an easy-to-use assay, allowing monitoring of virus penetration, independent of other stages of virus entry or replication in a medium- or high-throughput fashion.

Presented here is a versatile assay usable in different formats to allow distinctive analysis of the viral penetration/fusion process, as well as binding and internalization of viral particles in a replication-independent manner. They use minimal enzyme complementation of the well-studied *E. coli* enzyme β-galactosidase. Enzyme activity is reconstituted when a small peptide (α-peptide) is paired with an inactive mutant form of β-galactosidase (ΔM15), lacking residues 11-41 of the lacZ β-galactosidase [26]. The 45aa α-peptide, representing aa 5–51 of the lacZ β-galactosidase, is attached to either the C- or the N-terminus of an intravirion viral protein [27]. ΔM15 is expressed transiently or stably in the cytosol of target cells. When the spatial separation of the α-peptide in the virion and ΔM15 is removed, for instance when viral and cellular membrane fuse, complementation can be detected.

We have established and tested the method for two different enveloped viruses: murine hepatitis coronavirus (MHV strain A59, further referred to as MHV), which belongs to the *Coronaviridae*, and vesicular stomatitis virus (VSV) belonging to the *Rhabdoviridae*. Coronaviruses (CoVs), plus-stranded RNA viruses, infect a variety of mammals and birds. They include important pathogens, such as SARS-CoV [28] and MERS-CoV [29], which cause severe respiratory tract diseases in humans. VSV is a negative-sense RNA virus with a broad host spectrum, which ranges from mammals to insects. It regularly causes severe epidemics in livestock [30], [31]. VSV is a good model virus for this new method as its entry process has been well characterized [10], [30]. We generated α-peptide tagged MHV and VSV virions. By using enzymatic amplification the binding, internalization, and fusion of both viruses could be separately and efficiently measured at low multiplicity of infection (MOI).

## Materials and Methods

### Cells, viruses, and antibodies

Murine LR7 fibroblast [32] (based on murine L cells, orig. ATCC), feline FCWF (ATCC) and human HEK293T (ATCC) cells were used to propagate the viruses (i.e. recombinant MHV, interspecies chimeric coronavirus fMHV [32], and pseudotyped VSV, respectively). Cells were maintained as monolayer cultures in Dulbecco's modified Eagle's medium (DMEM, Lonza), supplemented with 10% fetal calf serum (FCS).

LR7 and HEK293T stably or transiently expressing ΔM15 in the cytosol have been used for infection experiments. Stable cell lines were generated using a Moloney murine leukemia (MLV) retroviral vector. MLV was produced in HEK293T cells by triple plasmid transfection of a transfer vector containing ΔM15 gene as well as a puromycin resistance marker gene, in combination with expression vectors encoding the MLV Gag-Pol, and VSVG spike protein, respectively. Upon MLV transduction, stably transduced cells were selected at 2 µg/ml puromycin, maintenance at 1 µg/ml puromycin (Sigma) in DMEM, supplemented with 10% FCS.

The rabbit polyclonal antisera K114 [33] and K135 [34] to VSV and MHV-A59, respectively, have been described before as is the mouse monoclonal antiserum 10G, which is directed against the MHV-A59 S2 domain [35].

### Chemicals

The MHV fusion inhibitor HR2 peptide has been described before [36] and was synthesized by GenScript. The peptide was diluted in Tris/HCl 50 mM, pH 7.8, 4 µM EGTA at 1 mM stock solution and used at 10 µM final concentration. Fluorescein-di-β-D-galactosipyranoside (FDG) (AnaSpec) was diluted in DMSO to a stock solution of 20 mM. Purified *E. coli* β-galactosidase (Sigma-Aldrich) was diluted to 1E-7 g/µl in 100 mM Sodium Phosphate Buffer, pH 7.3 immediately prior to use.

Stocks of 5-bromo-4-chloro-3-indolyl-β-D-galactopyranoside (X-Gal, Sigma) were prepared at 40 mg/ml in dimethyl sulfoxide (DMSO). Stocks of 500 mM potassium ferrocyanide (K_4_[Fe(CN)_6_), 500 mM potassium ferricyanide (K_3_[Fe(CN)_6_), and 200 mM magnesium chloride (MgCl, all Sigma) were prepared in water (H_2_O).

Stocks of 700 mM cycloheximide (CHX, Sigma), 125 µM bafilomycin A1 (BafA1, Enzo Life Sciences), 120 mM dynasore (Dyn, Enzo Life Sciences), 1 mM nocodazole (Noc, Sigma), 1 mM latrunculin A (LatA, Sigma), 2 mM jasplakinolide (Jasp, Sigma), 1 mM brefeldin A (BrefA, Sigma) were prepared in DMSO and used at 1∶1000 final concentration.

Stocks of 2 M ammonium chloride (NH_4_Cl, Fluka), 10 mM chlorpromazine (Chlopro, Sigma) were prepared in H_2_O and used at 1∶100, 1∶1000, and 1∶250 final concentration, respectively.

A stock of 6 mM monensin (Mon, Sigma) was prepared in methanol (MeOH) and used at 1∶1000 final concentration.

### Plasmids

The α-peptide cDNA was isolated from an *E. coli* field isolate by DNA extraction and PCR. The cDNA was subcloned into a pCAGGS vector by restriction/ligation (BamHI/SbfI) and used from there. The ΔM15 gene was isolated from a DH5α *E. coli* lab strain by DNA extraction and PCR. The gene was cloned into a pCAGGS vector for (transient) expression and into a MLV-based pQCXIP transfer vector (Clontech) for the generation of stable cell lines, by restriction/ligation (XmaI/NotI in pCAGGS, SmaI/PacI in pQCXIP).

The transcription vectors for the production of donor RNA for targeted interspecies recombination of fMHV were derived from pMH54 [32], [37]. Constructs containing S-α, or α-N fusion genes were made by overlap-extension PCR and cloned into the parental pMH54 vector by restriction and ligation (MluI/SbfI for S-α, XbaI/NheI for α-N), resulting in the pMH54-Sα and pMH54-αN vectors, respectively. The expression vector pCAGGS-VSVGα for producing pseudotyped VSVΔG/FLuc-Gα* or VSVΔG/GFP-Gα* viruses was cloned from a pCAGGS-VSVG vector by overlap extension PCR and cloning (restriction/ligation with SacI/NotI).

### Generation of recombinant (pseudo-) viruses

Recombinant MHV-αN and MHV-Sα viruses were generated by targeted RNA recombination as described before [32]. Briefly, donor RNA was generated from linearized transfer vectors, described above, and electroporated into FCWF cells infected with interspecies chimeric fMHV coronavirus (an MHV-A59 derivative, in which the ectodomain of spike has been replaced by a spike ectodomain of a feline coronavirus, thereby changing host cell tropism). The electroporated FCWF cells were seeded onto a monolayer of LR7 cells. After 24 h of incubation at 37°C, supernatant medium containing progeny viruses was harvested. Recombinant viruses were subjected to two rounds of plaque purification on either LR7 or LR7ΔM15, after which passage 1 stocks were grown. Genotypes of the recombinant viruses were confirmed in passage 1 stocks, passage 2 stocks were used in experiments.

Recombinant MHV-EGFPM was generated as described above using the transcription plasmid pXHEGFPM, containing a GFP expression cassette between the E and M genes, while lacking ORFs 2a, HE, 4a, 4b, and 5a.

Recombinant VSVΔG/GFP or FLuc-Gα* pseudovirus was generated as described before [38]. Briefly, target HEK293T cells were transfected with pCAGGS-VSVGα 24 h prior to infection. VSVGα expressing cells were inoculated at MOI = 0.01 with VSVΔG/GFP or FLuc-Gα* pseudovirus. Cells were washed thoroughly at 4 hpi. At 20 hpi, or upon visible cytotoxicity of the viral infection in ca. 90% of the cells, virus-containing supernatant was harvested. This procedure was repeated once more in order to get rid of any residual VSVΔG/GFP or FLuc-Gα* pseudovirus in the new virus stocks.

Viruses were stored in culture medium, supplemented with 25 mM HEPES or upon sucrose cushion purification in TN buffer (10 mM Tris-Cl, pH 7.4, 10 mM NaCl).

### Blue/White selection – plaque purification of recombinant viruses

Monolayers of LR7ΔM15 cells were inoculated with MHV-αN or MHV-Sα viruses at appropriate (or increasing) dilutions. After 2 h of incubation at 37°C in infection medium (DMEM, supplied with 2% FCS) the inoculum was removed. Cells were subsequently overlaid with a 1∶1 mixture of 3% purified Agar (Sigma) in H_2_O (previously prepared and autoclaved, reheated prior to use and kept at 42–50°C until use) and EMEM (Gibco) supplemented with 20% FCS, 200 IU/ml penicillin, and 200 µg/ml streptomycin (both Life Technologies), 5 mM K_3_[Fe(CN)_6_, 5 mM K_4_[Fe(CN)_6_, 2 mM MgCl, and 400 µg/ml X-Gal (EMEM solution pre-warmed to 37°C prior to use).

Infected and overlaid cells were incubated for up to two days at 37°C. Recombinant viruses, containing the α-peptide were selected based on the blue color of the plaques generated by these viruses. Blue cell plaques were excised and taken up in water and subjected to three freeze/thaw cycles, after which passage 1 stock was grown.

### Entry kinetics experiments

To determine their entry kinetics, MHV-EGFPM virus or VSVΔG/GFP-G* pseudovirus were bound to target cells in infection medium at 4°C for 90 min at MOI = 1 (after washing ca. 5–10% of cells got infected) to synchronize infection. Unbound virus was washed away with ice-cold PBS. Warm infection medium, containing 2% FCS, was added and cells kept at 37°C. At indicated time points post infection the medium was replaced by pre-warmed NH_4_Cl-containing infection medium. Virus infection was allowed to progress until 8 hpi upon which cells were harvested by trypsinization and fixed in 4% final concentration formaldehyde solution. Infection was quantified by FACS analysis on a FACS Calibur (Benson Dickson) using FlowJo software, 10’000 events of living cells were collected for each sample.

### Analysis of β-galactosidase activity in the fusion assay

Virus was bound to target cells in infection medium at MOI = 10 (unless indicated otherwise) to synchronize infection for 90 min at 4°C. After synchronization cells were shifted to 37°C to allow infection for a suitable amount of time (allowing fusion to occur but stopping before virus is being degraded; 40 min for VSV, 90 min for MHV). To stop infection and harvest, cells were washed with cold trypsin-EDTA (Gibco), containing 25 mM HEPES and NH_4_Cl (to stop infection from progression). NH_4_Cl-containing trypsin was added and cells were incubated on ice for 30 min. Cells were resuspended with 5%FCS in PBS and transferred into cold 2 ml Eppendorf tubes. Cells were collected by centrifugation at 450 rcf for 5 min at 4°C and, after removal of supernatant, resuspended in 100 µl room-temperature 5%FCS/PBS. Immediately 100 µl room-temperature FDG (at 200 µM in H_2_O) was added. This induced a hypotonic shock enabling the uptake of the FDG substrate. After 3 min cells were rescued by adding an excess of ice-cold 5%FCS/PBS. Cells were again collected by centrifugation at 450 rcf for 5 min at 4°C, followed by removal of supernatant after which the cells were resuspended in 100 µl ice-cold 5%FCS/PBS and transferred into FACS tubes. FDG loaded cells were incubated on ice for 8–16 h (unless otherwise indicated 14 h, for details see **Fig. S5** in File S1) and analyzed by FACS. While this was the preferred protocol for our investigations of the effect of endocytosis affecting agents on viral entry, we also developed an alternative method to pre-load cells with FDG prior to treatment, where needed, and then infecting the cells.

Therefore, the supernatant of adherent target cells was removed and replaced by a 1∶1 mixture (room temperature) of 5%FCS/PBS: 200 µM FDG/H_2_O. After 3 min incubation at room-temperature an excess of 5%FCS/PBS was added, supernatant removed and replaced by growth medium. Cells were allowed to recover for 30 min at 37°C before further treatment (infection, drug treatment, etc.) was undertaken. Binding and infection were carried out as described above. Cells were harvested after 2 h of infection (allows fluorescein signal to build up) by trypsinization with trypsin-EDTA (containing 25 mM HEPES and NH_4_Cl) at 37°C for 10 min. Cells were harvested with ice-cold 5%FCS/PBS and transferred to Eppendorf tubes, collected by centrifugation at 450 rcf for 5 min at 4°C, supernatant removed and cells resuspended in 100 µl ice-cold 5%FCS/PBS, after which they were immediately analyzed by FACS. This was our protocol of choice for investigations of more long-lasting agents/treatments, such as siRNA and dn/ca construct transfection.

For adherent cell microscopy analysis cells may be infected without pre-loading as described above. After the appropriate infection time supernatant is removed and replaced by a 1∶1 mixture (room temperature) of 5%FCS/PBS: 1 mM FDG/H_2_O. Upon a 3 min incubation at room-temperature an excess of ice-cold 5%FCS/PBS is added to stop the hypotonic shock. The supernatant is removed and the cells overlayed with ice-cold 5%FCS/PBS. The samples are incubated at 4°C for 8–16 h. Cells are allowed to recover and flatten by incubation at 37°C for 30 min before they are analyzed by microscopy. Cells were analyzed using an EVOS inverted fluorescence microscope.

Cycloheximide may be added to the cells to prevent viral protein synthesis. Microscopy analysis is also compatible with a pre-loading protocol.

### Western blotting

For western blotting of viral structural proteins the viruses were purified and concentrated over a 20% Sucrose (in TN buffer) cushion at 75’000 average rcf. Pelleted virus was resuspended in TN buffer overnight at 4°C, SDS loading buffer added to a final concentration of 100 mM DTT, boiled for 5 min at 95°C and subjected to western blotting in 7% acrylamide (37.5∶1, Bio-Rad) gels. Upon transfer to a nitrocellulose membrane (Millipore) the viral proteins were probed with antibodies K135 (rabbit anti-MHV pAb), 10 G (mouse anti S2 mAb), and K114 (rabbit anti-VSV pAb) on MHV-αN, MHV-Sα, and VSVΔG/GFP or FLuc-Gα*, respectively (all 1∶1000). Blots were developed using Rabbit anti-mouse HRP or Swine anti-rabbit HRP (both 1∶5000,DAKO).

To analyze intracellular virus protein, infected cells were harvested as described above for the entry assay. Due to the trypsin treatment cell-bound virus was removed and only intracellular virus remained. Half of the cells were then subjected to FDG treatment and β-galactosidase activity measurement, whereas the other half was mixed with SDS loading buffer and subjected to western blotting as described above. GM130 (rabbit monoclonal, Abcam) antibody was used as loading control detection.

### Analysis of β-galactosidase activity in the binding and internalization assay

Virus was bound to the target cells at MOI = 10 (unless otherwise indicated) for 90 min at 4°C.

For the binding assay unbound virus was washed away with ice-cold PBS. The cells and viruses were subsequently lysed with NP-40 lysis buffer (50 mM Tris/HCl pH 8.0, 150 mM NaCl, 0.5% NP-40) supplemented with Complete Protease Inhibitor Cocktail (Roche) for 10 min at room-temperature. An appropriate amount of the lysate was transferred into a luminometer plate and supplemented 1∶1 with 100 mM Sodium Phosphate Buffer, pH 7.3. After transfer to the Centro LB 960 luminometer (Berthold technologies) 30 µl/well Beta-Glo reagent (Promega) was added to each well, the sample was mixed and incubated for 30–210 min and light units were measured over 0.1 second.

For the internalization assay unbound virus was washed away with ice-cold PBS after a short heat shock at 37°C for 1 min, warm infection medium was added and cells shifted to 37°C for an appropriate amount of time (30 and 80 min for VSV and MHV, respectively). Cells were trypsinized to remove surface bound but not internalized virus. Cells were resuspended in ice-cold 5% FCS/PBS and immediately collected by centrifugation at 450 rcf for 5 min at 4°C. Supernatant was removed and the cell pellet resuspended in lysis buffer. β-galactosidase activity was measured as described above for the binding assay.

To generate calibration curves we used either purified *E. coli* β-galactosidase diluted in 1∶1 NP-40 lysis/100 mM Sodium Phosphate buffer, pH 7.3 or sucrose cushion purified virus resuspended in TN (analyzed for infectivity) and lysed by NP-40 lysis buffer.

### Infection assays

For all infection assays target cells were pre-treated with drugs if indicated for 30 min at 37°C prior to virus binding. Subsequent binding, internalization, and fusion (as far as needed for the respective assay) were carried out in presence of the drugs at indicated concentrations (see chemicals section).

### Growth curves of recombinant viruses

LR7 cells were infected at MOI = 0.5 of the respective virus (MHV-A59 wt, MHV-αN and MHV-Sα viruses) in infection medium containing 25 mM HEPES. After 3 h of infection supernatant was replaced by fresh infection medium and infection was allowed to progress over a period of 24 h. Every 3 h a small sample of the supernatant was collected and immediately frozen. The supernatant samples were subsequently analyzed in TCID50 assays on LR7 cells.

### Electron microscopy

VSVΔG/GFP-G* or VSVΔG/GFP-Gα* pseudovirus was purified through a sucrose cushion as described before. Virus was prepared as described before [39]. Briefly, pelleted virus was resuspended in 50 mM Tris-HCl, pH 7.5 with 100 mM NaCl buffer or in 50 mM MOPS, pH 6.6 with 100 mM NaCl buffer. The pH 6.6 dissolved virus was incubated for 15 min at 37°C and subsequently dialyzed at room temperature against 50 mM MOPS, pH 5.5 with 100 mM NaCl buffer for 30 min. The virus preps (pH 7.5 and pH 5.5) were adsorbed onto a discharged carbon film and subjected to negative staining (2% uranyl acetate solution). Probes were analyzed with a Philips CM200 microscope at 100 kV.

## Results

### Outline of the replication-independent entry assays

**Figure 1:**
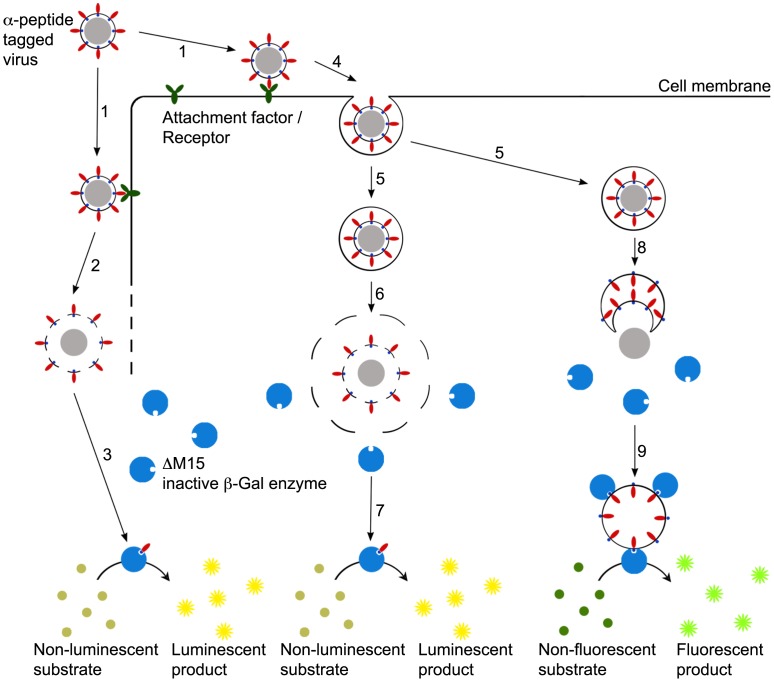
Design of the virus entry assays. Schematic overview of binding- (left), internalization- (middle), and fusion assay (right). 1 - Binding of virus to cell membrane; 2 – Lysis of cells and surface-bound virus; 3 – Complementation of ΔM15 by intravirion α-peptide, substrate conversion yielding luminescent readout; 4 – Invagination and 5 – Budding of endosomal vesicles containing virus particles; 6- Lysis of cell, intracellular compartment, and virion (after removal of cell surface-bound virions by protease treatment); 7 - Complementation of ΔM15 by intravirion α-peptide, substrate conversion yielding luminescent readout; 8 – Fusion of virion with endosomal membrane, exposure of intravirion α-peptide to the cytosol; 9 – Complementation of intracellular ΔM15 by virion α-peptide in intact cells, substrate conversion yielding fluorescent readout.

Based on minimal complementation of β-galactosidase we devised three assay formats to enable the differential analysis of cell binding, internalization, and fusion of viruses. In the binding assay recombinant viruses containing the α-peptide as an intravirion protein tag (α-viruses) are allowed to bind to the surface of target cells on ice. After removal of unbound virus the amount of bound virus particles is quantified by enzyme complementation upon lysis of cells and their attached virions, ΔM15 being provided either by expression in the target cells or by including it in the lysis buffer. Complementation is detected using a sequential system of substrate conversion by β-galactosidase and luciferase to generate a luminescent signal (**Fig. 1**, left). In the internalization assay the previously surface-bound α-viruses are allowed to enter cells by warming to 37°C. Surface-bound but not internalized virus particles are removed by protease treatment (e.g. Trypsin or Proteinase K) on ice prior to lysis of cells and internalized virions. This is followed by measurement of complementation as described before (**Fig. 1**, middle). The fusion assay is based on analysis of intact cells. Thus, α-virus is bound to cells expressing ΔM15 and allowed to enter at 37°C. The spatial separation of the α-peptide and ΔM15 is not lifted by lysis but by fusion or penetration of the α-virus. Subsequently, the activity of the complemented β-galactosidase is quantitated by measuring its degradation of the non-fluorescent substrate fluorescein-di-β-D-Galactopyranoside (FDG) into green fluorescent fluorophores fluorescein (**Fig. 1**, right).

### Attachment of the α-peptide to viral proteins and validation of complementation

**Figure 2:**
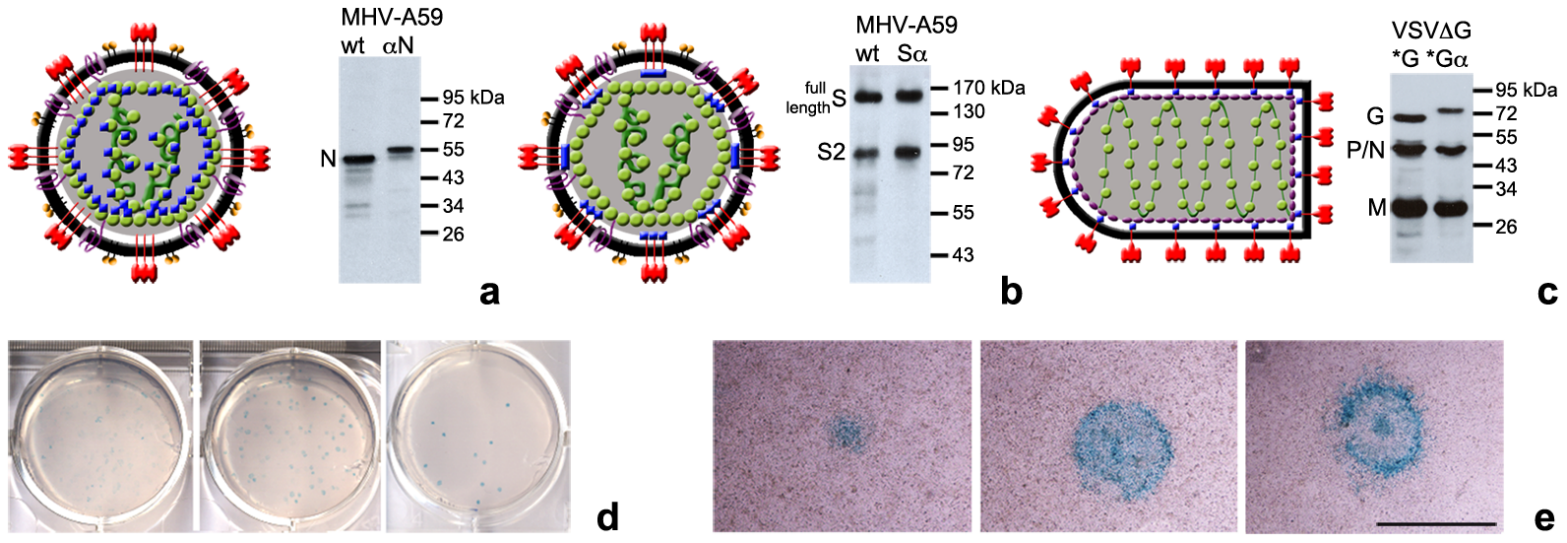
Model of viruses carrying α-peptide tagged proteins and visual selection of recombinant viruses and plaque growth by α-complementation. (**a–c**) α-peptide is shown as blue squares. (**a**) Model of MHV-αN and western blot analysis of N protein in purified virus stock. (**b**) Model of MHV-Sα and western blot analysis of S protein in purified virus stock. (**c**) Model of VSVΔG-Gα* pseudovirus and western blot analysis of VSV structural proteins in purified virus stock. (**d**) Serial dilution plaque assay of recombinant MHV-αN on LR7ΔM15 cell monolayers. After inoculation cells were covered for 2 days with a X-Gal containing agar-medium overlay. (**e**) Visualization of plaque growth of MHV-αN in LR7ΔM15 cell monolayers after 16, 30 or 48 h incubation (from left to right). Size bar corresponds to 1 mm.

To investigate the possibilities and consequences of the integration of α-peptide into virions we generated MHV and VSV derivatives carrying α-peptide-tagged structural proteins. Thus, recombinant MHV were obtained with the α-peptide fused either to the C-terminus of the spike protein (Sα) or to the N-terminus of the nucleocapsid protein (αN) (**Fig. S1** in File S1). We pre-tested the complementation assay by transient co-expression of the tagged proteins with the ΔM15 protein in HEK293T cells, which confirmed that both fusion proteins efficiently complemented the defective galactosidase ΔM15 as shown for the αN protein in **Figure S2a** in File S1. The recombinant viruses were generated by homologous targeted RNA recombination [37]. Their growth properties were affected slightly by the addition of the α-peptide tag to the N or S protein. The impact on growth of MHV-αN seems to be merely a delay in growth. The decrease of viral yield for MHV-Sα is significantly lower but within comparable margins for other recombinant MHV viruses with modified spike proteins (**Fig. S3** in File S1). Analysis of their structural proteins by western blot showed the predicted weight shift of 5 kDa for the α-peptide tagged N protein. Due to its larger size and heterogeneous glycosylation, a shift in electrophoretic mobility was not clearly visible for the MHV-Sα protein (**Fig. 2a and b**). The genetic identity of the recombinant coronaviruses was confirmed by sequence analysis.

To demonstrate complementation in infected cells and to devise a potential new way of selecting recombinant (MHV) viruses we adapted the blue/white screening method generally used for the selection of transformed bacterial colonies [40]. ΔM15-expressing cells infected with recombinant virus were overlaid with an agar-medium mixture containing 5-bromo-4-chloro-indolyl-β-D-galactopyranoside (X-Gal). Degradation of X-Gal by β-galactosidase yields a blue colored precipitate (5,5′-dibromo-4,4′-dichloro-indigo). Indeed, recombinant viruses containing the α-peptide fusion protein generated blue plaques. Performing blue/white screening provided a convenient way to score plaque assays by eye and simplified the selection and purification of recombinant viruses (**Fig. 2d**). X-Gal did not appear to harm the target cells, even when treated for several days, which allowed us to monitor plaque growth and viral spread in live cells (**Fig. 2e**).

As a second model system, we chose pseudotyped VSV (described by Tani et al. [38]). VSV lacking the G-gene in its genome was complemented with a C-terminally tagged VSV-Gα protein expressed in cells used to produce the pseudotyped VSV (**Fig. S1** in File S1). In the genome of the pseudovirus the G gene was substituted by the gene for firefly luciferase or green fluorescent protein (GFP). Western blot analysis of structural virus proteins demonstrated the characteristic 5 kDa shift in electrophoretic mobility of Gα protein due to the addition of α-peptide (**Fig 2c**). VSV pseudotyped with the Gα (VSVΔG-Gα*) amplified to similar titers as its wild type counterpart (VSVΔG-G*; data not shown). Incorporation of VSV-Gα was confirmed by EM. Functionality of these proteins was confirmed by low pH treatment of pseudotyped particles, which resulted in the previously observed structural rearrangement of the spikes on the virion surface (**Fig. S4** in File S1) [39].

### Virus-cell fusion assay

**Figure 3:**
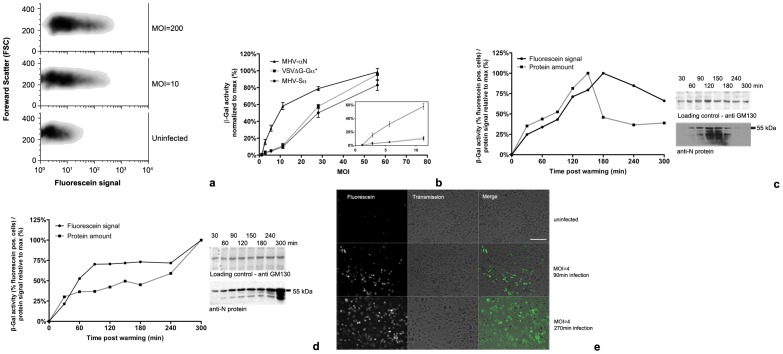
Fusion assay. (**a**) Virus-cell fusion measured by flow cytometry. Sorting of MHV-αN infected cells by flow cytometry showed increasing fluorescence at increasing MOI. Cells were treated as described in **b**. (**b**) Increase of fusion signal relative to MOI. Increasing amounts of MHV-αN, MHV-Sα, and VSVΔG-Gα* were bound to ΔM15 expressing cells on ice. 40 min (VSV) or 100 min (MHV) post warming to 37°C fusion was assayed by measuring β-galactosidase activity using FDG substrate and flow cytometry. Inlay highlights β-galactosidase activity at low MOI. Error bars represent 1 SEM, n = 3. (**c, d**) Kinetics of internalized α-peptide tagged protein in comparison to β-galactosidase activity. MHV-αN (MOI = 100) was bound to cells on ice. Unbound virus was removed, and samples shifted to 37°C with (**c**) or without addition of cycloheximide (**d**). At the indicated time points, cells were washed and trypsinized on ice, removing surface bound virus. Virus-cell fusion was measured by β-galactosidase activity using flow cytometry or cells were lysed and immunoblotted against N for quantification the internalized α-peptide proteins. (**e**) Fluorescence microscopy image of β-galactosidase activity in infected cells. MHV-αN was bound to LR7ΔM15 cells on ice. Inoculum was washed off and cultures shifted to 37°C for the indicated time periods. β-galactosidase activity was visualized by fluorescein production using fluorescence microscopy. Size bar corresponds to 250 µm.

Our initial aim was the establishment of the virus-cell fusion assay. Fusion of the viruses containing α-peptide-tagged structural proteins with host cells was assessed by inoculation of ΔM15 expressing cells with increasing concentrations of purified, concentrated α-virus. To synchronize infection MHV-αN, MHV-Sα, and VSVΔG-Gα* were allowed to bind to LR7 or HEK293T cells expressing ΔM15 for 90 min on ice. Unbound virus was then removed and cultures were shifted to 37°C and incubated for 100 (MHV) or 40 min (VSV), based on earlier studies of virus entry kinetics (**Fig. S5** in File S1). Incubation was stopped by cooling the cells to 4°C. Cells were detached on ice and FDG substrate was added in combination with a short hypotonic shock, which results in pinocytic uptake of FDG [41]. Similar results were obtained when cells were pre-loaded with substrate (data not shown, see methods for procedure). To prevent protein degradation and further progression of the infection, resulting in expression of new viral proteins, cells were continuously kept on ice. As low temperatures slow down the enzymatic activity of β-galactosidase, prolonged incubation was required to obtain a strong fluorescein signal. After MHV-αN inoculation at MOI = 10, a maximum fluorescein signal was reached after 10 h incubation on ice, which remained stable for 24 h (**Fig. S6** in File S1). Inoculation at increasing MOI resulted in increased β-galactosidase activity, as measured by flow cytometry. The cell density plots show that the maximum fluorescence is equal in low and high MOI infections (**Fig. 3a**). The median fluorescence shifts to higher values at higher MOI. With this fusion assay significant increases in fluorescein signals were obtained already at MOIs around 2–4 for MHV-αN and at MOIs of 5–10 for MHV-Sα and VSVΔG-Gα* (**Fig. 3b**). In all subsequent fusion assays an MOI of 10 was used and, because of its stronger signal, MHV-αN rather than MHV-Sα was the tagged MHV of choice.

In order to confirm that β-galactosidase activity depends on the presence of the α-peptide in the cells we assessed the correlation between the intracellular presence of tagged protein and fluorescein signal. Therefore, LR7ΔM15 cells were inoculated with MHV-αN as described above. Cells were detached by trypsinization, which also removed cell surface-bound viruses. Cells were analyzed by the fusion assay as described above. In parallel samples, cells were lysed and the intracellular viral αN protein content was determined by immunoblotting against N. The presence of intracellular αN protein correlated with the fluorescein signal generated by β-galactosidase activity. In the presence of the protein synthesis inhibitor cycloheximide (CHX), the signals peaked at 150 or 180 min (**Fig. 3c**). In absence of CHX the signals leveled off after 90 min and increased strongly after 300 min of infection, consistent with viral gene expression (**Fig. 3d**). Similar results were obtained for VSVΔG-Gα* (**Fig. S7** in File S1).

The β-galactosidase activity in infected cells was also visualized by fluorescence microscopy. LR7ΔM15 cells were inoculated with MHV-αN as described above. At 90 or 240 min post infection FDG was added to the cells. Samples were incubated for 14 h on ice and analyzed by fluorescence microscopy. Infection for 90 min generated fluorescent signals in the target cells. Prolonged infection, allowing replication-dependent increase of αN levels, resulted in increased fluorescent signals (**Fig. 3e**). Similar results were obtained for VSVΔG-Gα* (**Fig. S8** in File S1).

### Virus binding and internalization assays

**Figure 4:**
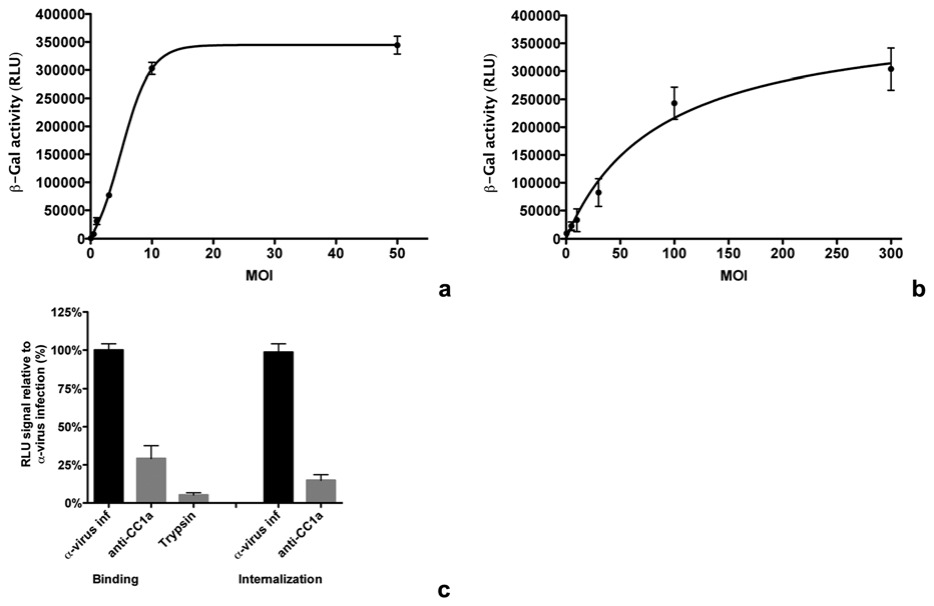
Binding and internalization assay. (**a**) Luminescent signal after virus binding at various MOI. Increasing amounts of MHV-αN were bound to LR7ΔM15 cells on ice for 90 min before removing the inoculum and washing-off of unbound virus with ice-cold PBS. Cells and bound viruses were lysed and binding was determined by measuring the β-galactosidase activity using Beta-Glo substrate conversion to a luminescent product. (**b**) Internalization signal relative to MOI. Increasing amounts of MHV-αN were bound to LR7ΔM15 cells on ice for 90 min. Inoculum was removed and samples transferred to 37°C for 40 min. Cell-surface bound virus was removed by trypsinization. Cells and intracellular viruses were lysed and internalization determined by measuring β-galactosidase activity using Beta-Glo substrate conversion to a luminescent product. (**c**) Controls of binding and internalization assay. Samples were treated as described in **a** (binding) and **b** (internalization). After binding, attached virus was removed by trypsin treatment (trypsin). Binding and internalization were inhibited by incubation of cells with MHV receptor CC1a blocking anti-CC1a antibody (anti-CC1a) 30 min prior to and during inoculation. Error bars in **a** - **c** represent 1 SEM, n = 3.

Binding and internalization of the α-peptide carrying viruses were assessed in assays, in which cells expressing ΔM15 were lysed after virus binding or internalization to allow complementation. For an initial characterization of virus binding, LR7ΔM15 cells were inoculated with increasing concentrations of purified, concentrated MHV-αN. Cells were overlayed with virus inoculum for 90 min on ice before removing unbound virus. Cells and viruses were lysed and incubated with Beta-Glo substrate, which allows a luminescent read-out of the β-galactosidase activity. Incubation for 50 min with the substrate at room temperature was optimal for measuring low β-galactosidase activities (data not shown). Lysis buffer did not interfere with the activity of the β-galactosidase (data not shown). Binding at very low MOI already increased the luciferase signal significantly. The half maximum value was reached approximately at MOI = 2 and a plateau was reached above MOI = 10 (**Fig. 4a**).

For the internalization assay LR7ΔM15 cells were inoculated with different concentrations of purified, concentrated MHV-αN. Cells were overlayed with virus inoculum for 90 min on ice before removing unbound virus. Virus was allowed to internalize by incubation at 37°C for 60 min. Cell surface bound virus was removed by trypsinization before cells and viruses were lysed. Samples were incubated with Beta-Glo substrate and β-galactosidase activity was measured as described above. The half maximum value was reached at MOI∼80 (**Fig. 4b**). A β-galactosidase standard allows the quantification of complemented enzymes corresponding to virus particles that fused or were present upon lysis, an example of which is shown in **Figure S9** in File S1.

To assess the specificity of the binding and internalization assays MHV-αN was bound to LR7ΔM15 cells in the absence or presence of anti-murine Ceacam1a (CC1a) antibody, which blocks the CC1a entry receptor used by MHV [42], and treated as described above for the binding assay. As a control, cell surface-bound virus was removed using trypsin before lysis. Trypsinization and blockage of the receptor dramatically decreased virus binding. Also virus internalization was inhibited by CC1a-antibody-dependent receptor blockage (**Fig. 4c**).

### Effect of inhibitors on distinct phases of VSV and MHV entry

To functionally assess the applicability of the entry assays we determined the effect of inhibitors on different entry stages of MHV and VSV. Prior to inoculation, ΔM15 expressing target cells were treated with inhibitory agents for 30 min at 37°C, and the same inhibitors were kept present throughout the experiment. The different stages of virus entry were assessed as described above.

**Figure 5:**
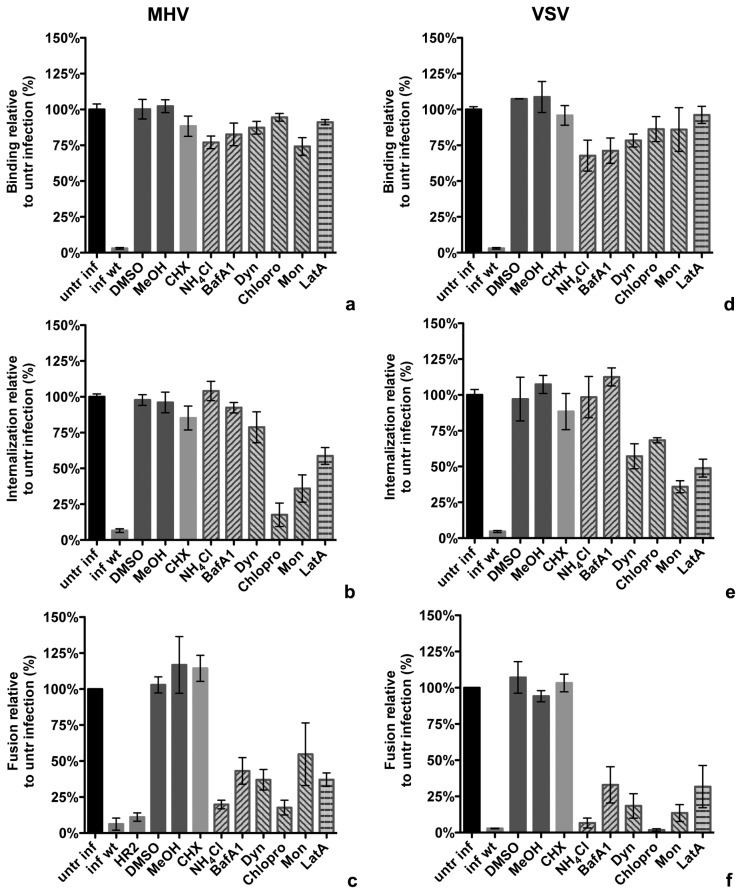
Effects of drugs on binding, internalization, and fusion of MHV and VSV. (**a–f**) Cells were pretreated with cycloheximide (CHX), ammonium chloride (NH_4_Cl), bafilomycin A1 (BafA1), dynasore (Dyn), chlorpromazine (Chlopro), monensin (Mon), or latrunculin A (LatA), as well as with solvents dimethyl sulfoxide (DMSO) and methanol (MeOH) for 30 min. MHV and VSV viruses without α-peptide were included as background controls (inf wt). Error bars represent 1SEM, n = 3. (**a, d**) MHV-αN or VSV-Gα* were bound to ΔM15 expressing cells in presence of compounds on ice for 90 min. Cells were washed, lysed and assayed with Beta-Glo substrate as described in **4a**. Binding was determined relative to the complementation luminescence signal generated by virus bound to ΔM15 cells, treated without compound added (untr inf). (**b,e**) After binding as described in **a**, MHV-αN and VSV-Gα* were allowed to internalize at 37°C in presence of compounds for 40 and 30 min, respectively. Internalization was determined relative to the complementation luminescence signal of virus internalized into ΔM15 cells, treated without compound added (untr inf). (**c,f**) After binding as described in **a**, MHV-αN or VSV-Gα* were allowed to internalize and fuse at 37°C in presence of compounds for 100 and 40 min, respectively. MHV fusion inhibitor HR2 peptide (HR2) was included as control. Fusion was determined relative to the number of positive cells showing complementation fluorescein signal of virus fused in ΔM15 cells, treated without compound added (untr inf).

Infection with non-α-peptide containing (wt) virus and solvents dimethyl sulfoxide (DMSO) and methanol (MeOH) were included as controls. Treatment of cells with CHX did not affect binding, internalization or fusion of MHV or VSV. Inhibition of endosome maturation with ammonium chloride (NH_4_Cl) or bafilomycin A1 (BafA1) strongly reduced fusion of MHV and VSV, but did not have a significant effect on binding or internalization. Treatment with dynasore (Dyn), an inhibitor of vesicle scission factor dynamin-2 prevented fusion for both viruses, only partially affected internalization, and had no influence on binding. Interference with the assembly of clathrin-coated vesicles using chlorpromazine (Chlopro) strongly decreased internalization and fusion of both viruses, as did the ionophore monensin (Mon), a known inhibitor of VSV entry [43]. Actin destabilizing agent latrunculin A (LatA) lead to reduced fusion for VSV and MHV and also affected internalization of both viruses (**Fig. 5a–f**).

## Discussion

In this article we present a novel method to dissect viral entry. Using minimal complementation of the β-galactosidase enzyme we were able to detect low numbers of virus particles at any stage of the entry process independent of replication. The assay discriminates between virus binding and internalization and makes it possible to specifically detect and quantify those virus particles that underwent fusion with a host cell. Measuring virus fusion in live cells not only allows for quantitative analysis but also for sorting infected from non-infected cells thereby enabling re-culture these cells. This also allows for combination of the entry assays with replication-dependent reporter assays to investigate later stages of the viral life cycle. Integration of α-peptide into both model viruses was feasible and had limited influence on their viability, suggesting that this novel method can be applicable to other viruses, including non-enveloped viruses. Particularly for the latter viruses it has proven difficult to integrate bulky tags, while labeling of a surrounding lipid layer is not possible. Generally, every virus for which it has been shown possible to attach tags to intravirion structural proteins will be a good candidate for this assay. Using cytosolic expression of ΔM15 in target cells does limit the applicability of the assay to viruses fusing towards the cytosol. However, this could be changed by expressing ΔM15 as a fusion protein in a fusion protein target compartment. Unfortunately, the need to express ΔM15 in the target cells hampers the investigation of fusion in e.g. primary cells. For “native” cells, not expressing ΔM15, the assay can be used to investigate binding and internalization by supplying ΔM15 during or after lysis.

The entry assays confirm and extend current knowledge on virus entry of MHV and VSV. Using the fusion assay we confirmed clathrin-mediated endocytosis of VSV to depend on the actin cytoskeleton [10], [30]. Interestingly, the effect of inhibitory agents on MHV entry was very similar to VSV, indicating a comparable uptake mechanism for both viruses. Treatment of cells with chlorpromazine, which causes clathrin lattices to redistribute, affected virus internalization and fusion of both VSV and MHV. Dynasore severely reduced fusion of MHV, but hardly affected internalization of this virus. While dynasore inhibits endocytosis by inhibition of the vesicle scission factor dynamin-2, it does not prevent the formation of invaginations. Viral particles, especially MHV, which in comparison to VSV can be engulfed completely by endocytic vesicles of approximately 100 nm diameter, trapped in such invaginations are likely much less accessible for removal by trypsin.

The novel entry assays provide several advantages over conventional assays. Using enzymatic amplification of a tagged viral protein allows looking at viral entry events independent of gene expression. Drugs affecting replication in general, such as translation inhibitors, which will inadvertently affect viral gene expression, can be tested independently for their effect on virus entry. The enzymatically-amplified readout allows performing infections at physiologically relevant conditions. With complementation happening timely proximal to the membrane fusion event, the assay allows for more precise kinetic measurements on virus entry. Also it should become easier to dissect effects of mutations in virions on entry and/or replication. Furthermore, the enzymatic activity can be quantified by a variety of different methods, opening up opportunities for high-throughput analysis by FACS or by automated fluorescence microscopy. The fusion assay might be improved by using yet to be developed alternatives to the FDG substrate, the fluorescein product of which is photolabile. Importantly, as we demonstrated the entry assays can be used in combination with various methods to perturb cellular processes involved in viral entry, including the use of inhibitors, RNA interference, knock-out cells, and by expression of dominant-negative or constitutive-active proteins. Hence we expect them to facilitate research on virus entry significantly.

## Supporting Information

File S1 **Figure S1.** Schematic layout of the genome of recombinant viruses. **Figure S2.** Effect of drug treatment on FDG uptake. **Figure S3.** Growth curves of recombinant MHV-α viruses. **Figure S4.** Morphology of negatively stained VSVΔG/GFP-Gα virus at neutral and low pH. **Figure S5.** Virus entry kinetics of MHV and VSV as measured by their sensitivity to lysosomal tropic agent NH_4_Cl. **Figure S6.** Fluorescein signal dependence on the incubation on ice. **Figure S7.** Intracellular α-tagged protein level in relation to β-galactosidase activity. **Figure S8.** Fluorescence microscopy of β-galactosidase activity in α-virus infected cells. **Figure S9.** Calculating virus binding and internalization using a standard curve.
(PDF)
