# Supplementary material for: Dissecting Virus Entry: Replication-Independent Analysis of Virus Binding, Internalization, and Penetration Using Minimal Complementation of β-Galactosidase
Source: PLoS One. 2014 Jul 15;9(7):e101762. doi: 10.1371/journal.pone.0101762 (PMC4099126; doi:10.1371/journal.pone.0101762)
Supplement: File S1 — Figure S1. Schematic layout of the genome of recombinant viruses. Figure S2. Effect of drug treatment on FDG uptake. Figure S3. Growth curves of recombinant MHV-α viruses. Figure S4. Morphology of negatively stained VSVΔG/GFP-Gα virus at neutral and low pH. Figure S5. Virus entry kinetics of MHV and VSV as measured by their sensitivity to lysosomal tropic agent NH4Cl. Figure S6. Fluorescein signal dependence on the incubation on ice. Figure S7. Intracellular α-tagged protein level in relation to β-galactosidase activity. Figure S8. Fluorescence microscopy of β-galactosidase activity in α-virus infected cells. Figure S9. Calculating virus binding and internalization using a standard curve. (PDF) [file pone.0101762.s001.pdf]

# SUPPLEMENTARY INFORMATION

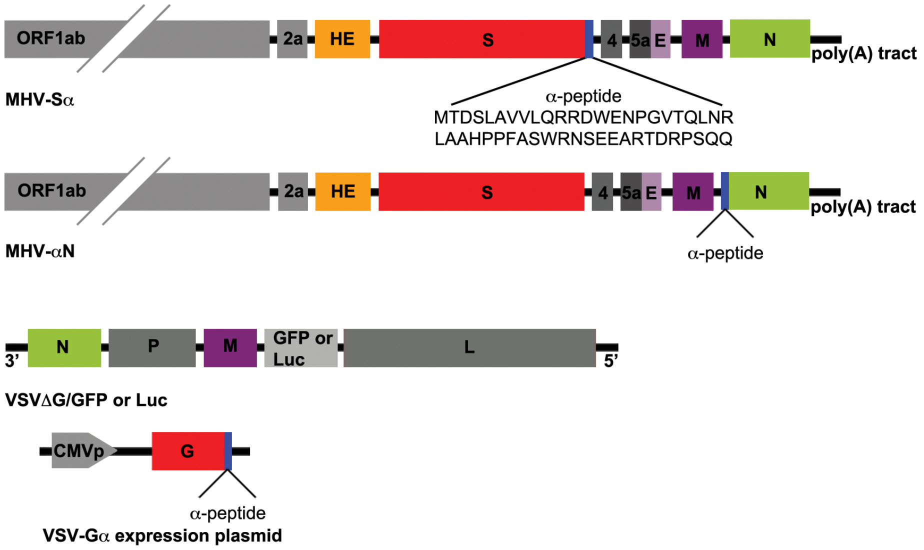


**Figure S1.** Schematic layout of the genome of recombinant viruses. MHV-Sα, encoding C-terminally α-peptide-tagged spike protein (top). MHV-αN, encoding the N-terminally α-peptide-tagged nucleocapsid protein (middle) and pseudotyped VSVΔG/GFP or -Luc virus coding a GFP or firefly luciferase expression cassette substituting the gene encoding the G protein. C-terminally tagged VSV-Gα is provided by the producer cells stably expressing VSV-Gα under the control of a CMV promotor.

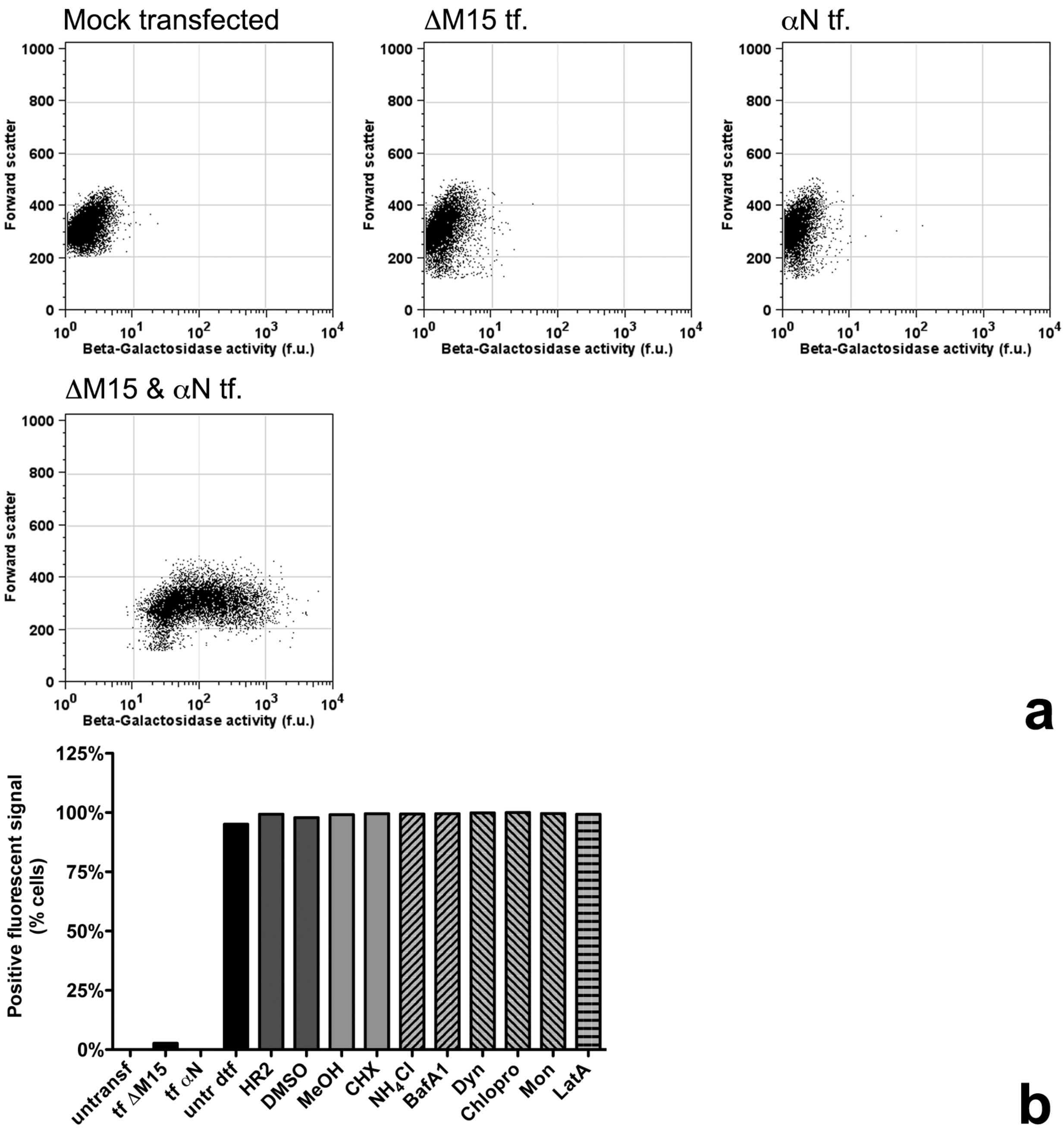


**Figure S2.** Effect of drug treatment on FDG uptake. (**a**) HEK293T cells were transfected singly or co-transfected with plasmids encoding ΔM15 or αN. Cells were trypsinized and FDG was added by hypotonic shock. Cells were incubated on ice for 1h and β-galactosidase activity measured by determining the production of fluorescein using FACS. (**b**) HEK293T cells co-transfected with ΔM15 and αN encoding plasmids were pretreated with the drugs used in the fusion assay (described in **5c** and **f**). Following pretreatment cells were trypsinized and FDG added by hypotonic shock. Cells were incubated on ice for 1h and β-galactosidase activity measured by determining the production of fluorescein using FACS.

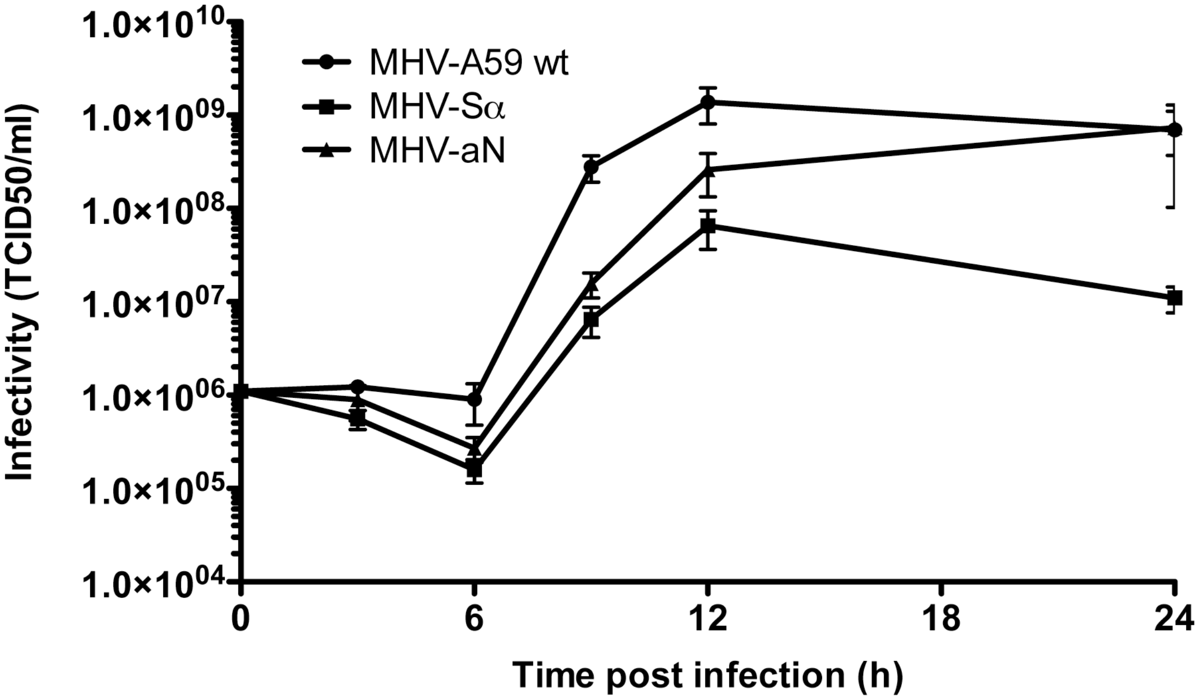


**Figure S3.** Growth curves of recombinant MHV-α viruses. Murine LR7 cells were inoculated at MOI=1 with the recombinant and wild-type A59 strain viruses, respectively. At the indicated times post infection, infectivity in the culture supernatants was assayed by TCID50 analysis. Error bars represent 1 SEM, n=4.

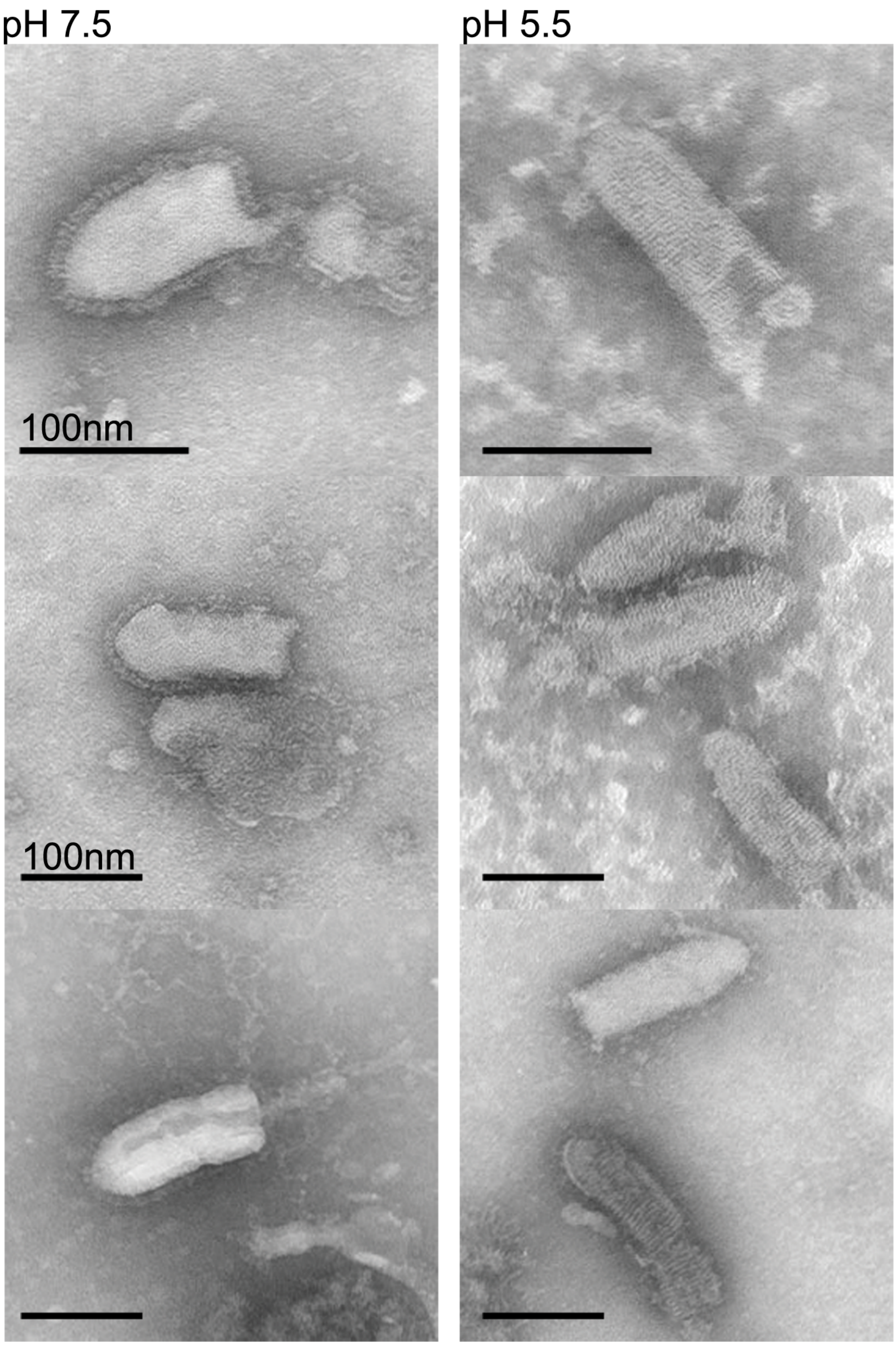


**Figure S4.** Morphology of negatively stained VSVΔG/GFP-Gα virus at neutral and low pH. 20% sucrose cushion purified stocks of VSVΔG/GFP-Gα, resuspended in TD buffer were sequentially brought to pH 6.5 and to the final pH of 5.5. Samples were negatively stained and imaged by electron microscopy. Three representative images for pH 6.5 and pH 5.5 are shown in the left or right panel, respectively.

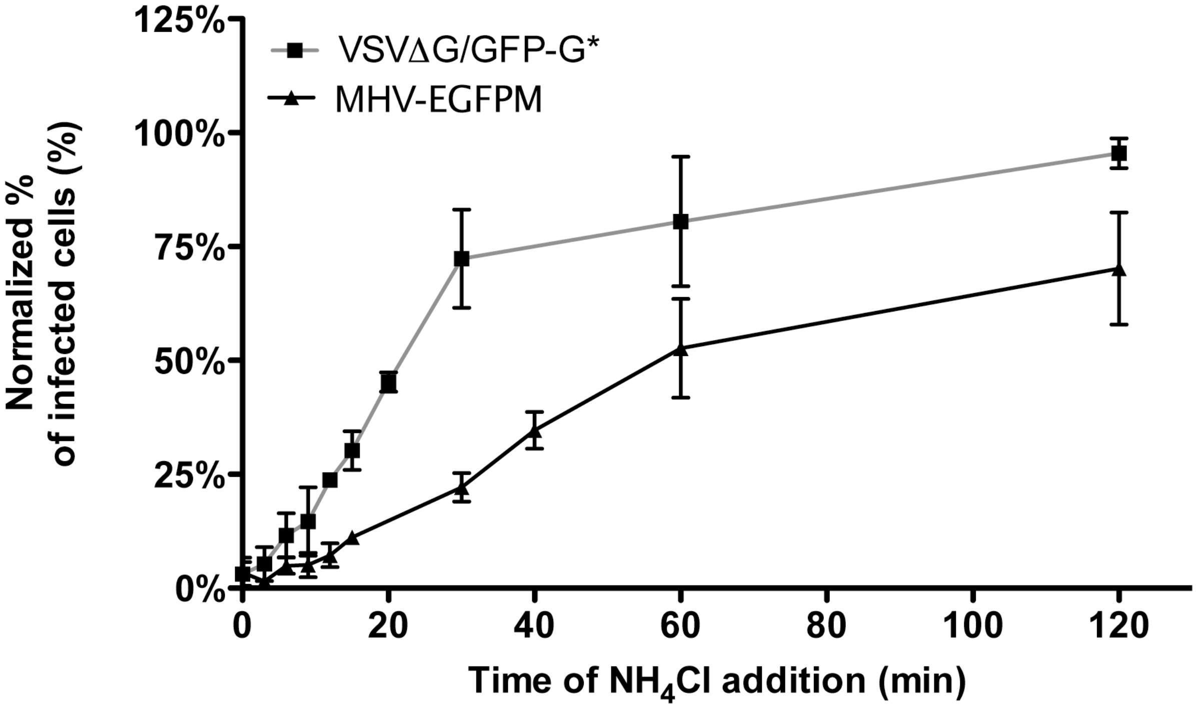


**Figure S5.** Virus entry kinetics of MHV and VSV as measured by their sensitivity to lysosomal tropic agent $NH_4Cl$. VSVΔG/GFP-G* and MHV-EGFPM were bound to cells on ice for 90 min at MOI=1. Unbound virus was removed with ice-cold PBS, and cultures were shifted to 37°C by adding warm medium. $NH_4Cl$ was added at the indicated time points to stop endosome maturation and thus virus entry. At 8h post warming, infection was scored by FACS analysis of GFP expression. Half maximum infection of VSV was obtained within 20 min, after 40 min approximately 80% of the maximal number of infected cells was obtained. MHV appeared to enter cells much slower and less synchronized. After about 50 min of warming only half maximum infection was obtained, after 100 min approximately 60% of the maximal number of infected cells was observed. Error bars in **a** & **b** represent 1SEM, n=3.

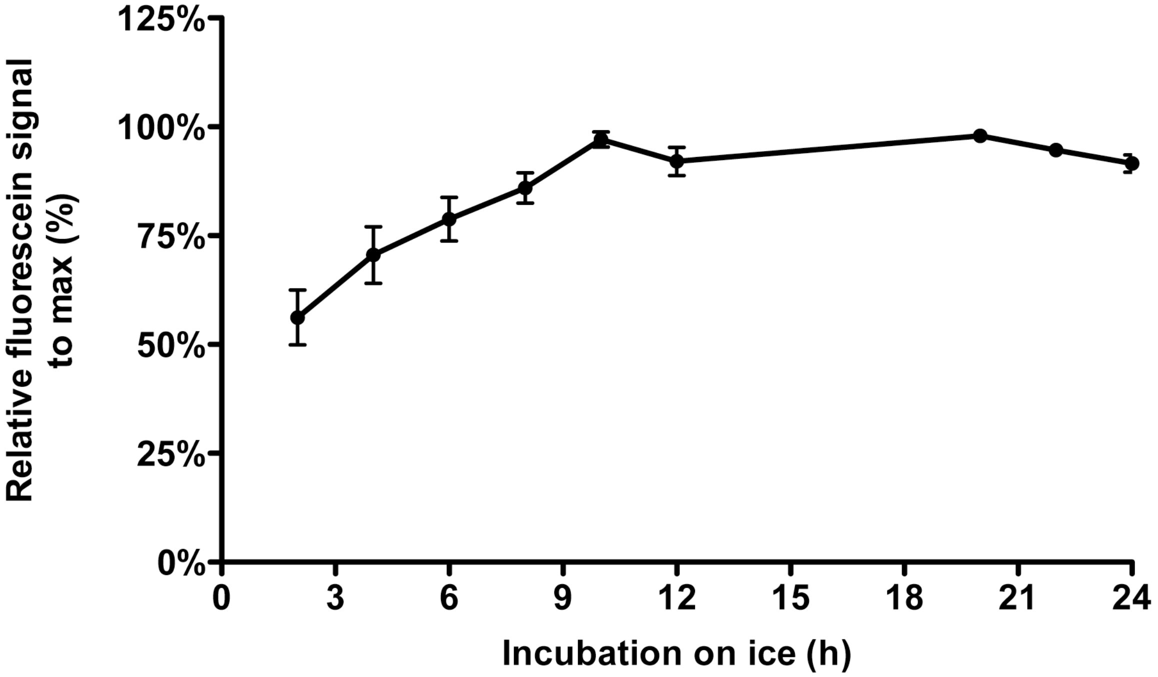


**Figure S6.** Fluorescein signal dependence on the incubation on ice. LR7ΔM15 cells were inoculated at an MOI of 10 with MHV-αN by binding the virus to the cells for 90 min on ice, removal of unbound virus, and shifting of the cultures to 37°C for 100min. FDG was administered by hypotonic shock and subsequently the samples were incubated on ice for the indicated amount of time. β-galactosidase activity was measured by determining fluorescein production using FACS.

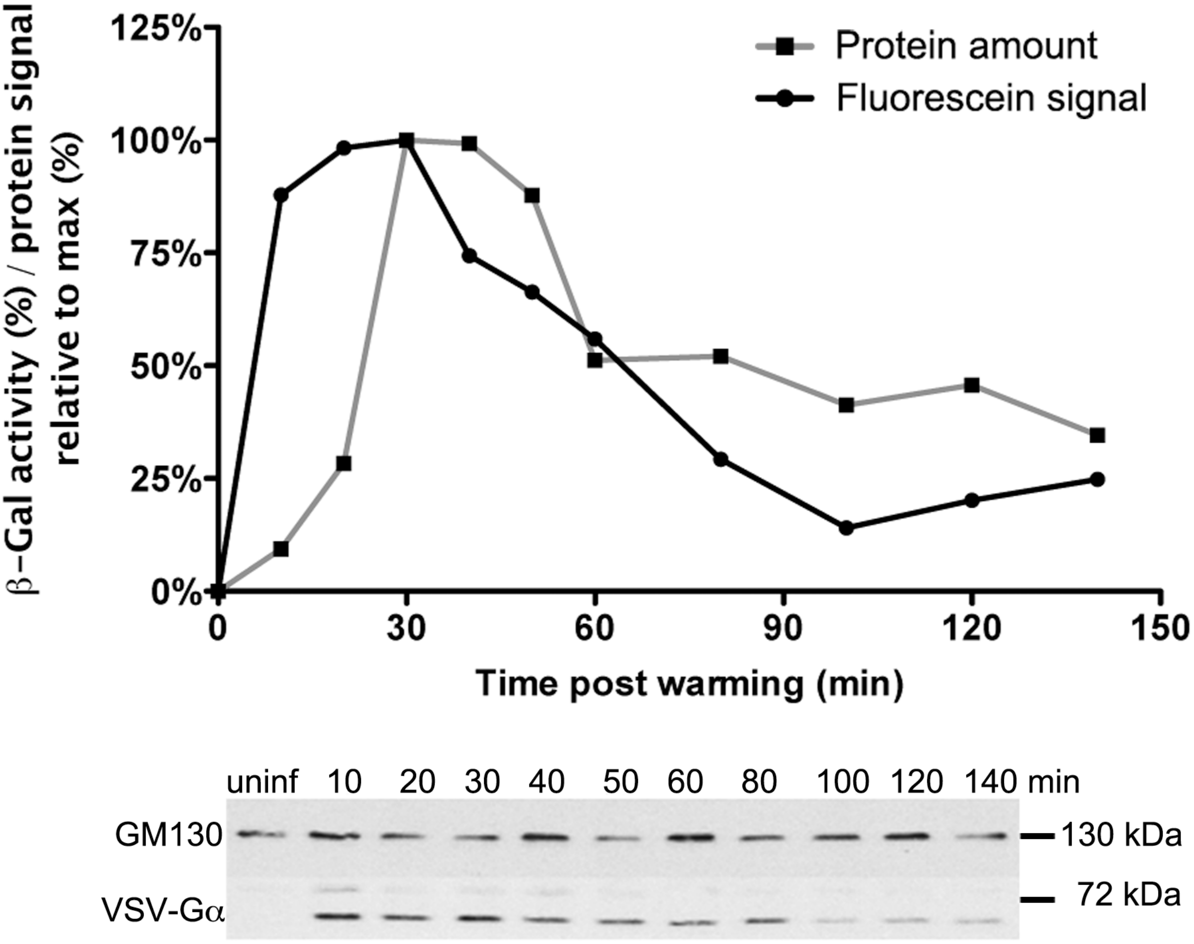


**Figure S7.** Intracellular α-tagged protein level in relation to β-galactosidase activity. VSVΔG/Luc-Gα* (MOI=100) was bound to cells on ice. Unbound virus was washed off, and culture temperature shifted to 37°C in the presence of CHX. At the indicated time points cells were washed and trypsinized on ice. Fusion was assessed by β-galactosidase activity-driven fluorescein production using FACS. In parallel samples cells were lysed and G-protein content was measured by western blot analysis.

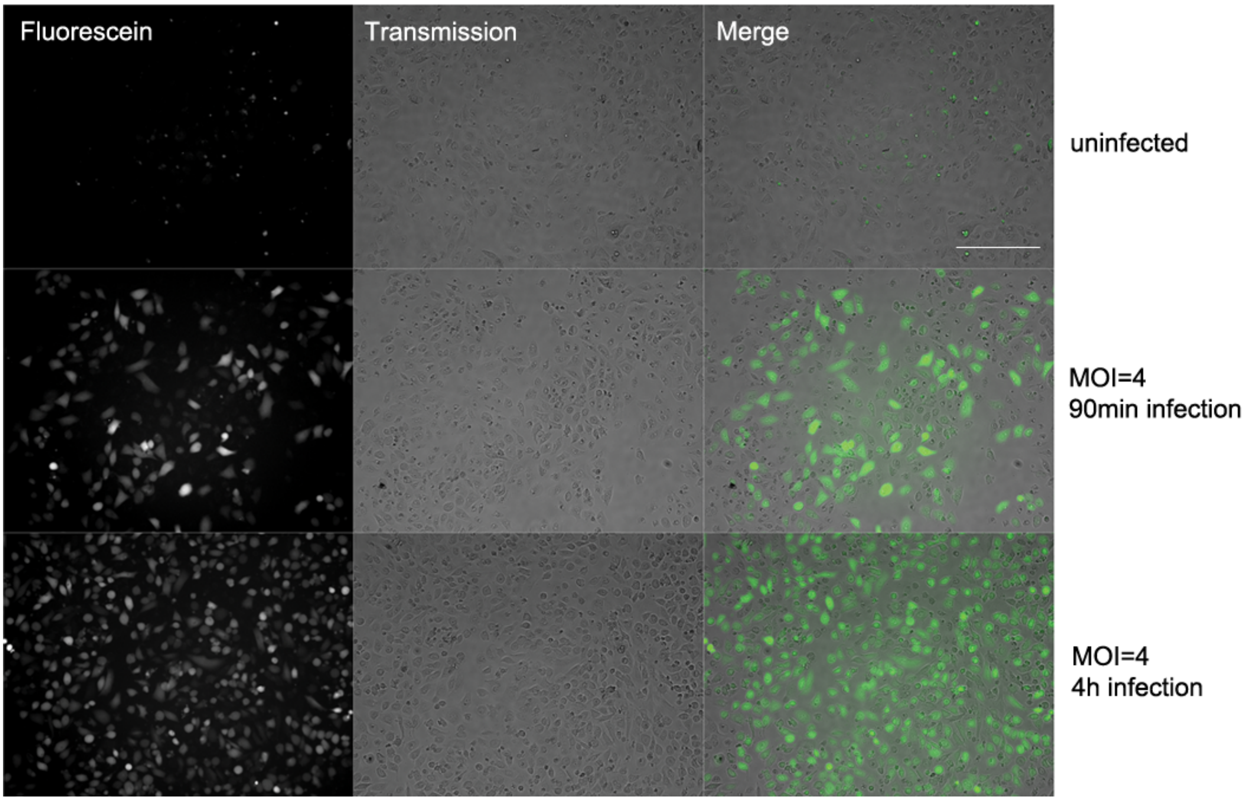


**Figure S8.** Fluorescence microscopy of β-galactosidase activity in α-virus infected cells. VSVΔG/Luc-Gα* virus was bound to ΔM15 transfected HEK293T cells on ice. Unbound virus was washed off and culture temperature shifted to 37°C for the indicated time periods. Fusion-dependent β-galactosidase activity-driven fluorescein production in cells was visualized by wide-field fluorescence microscopy. Size bar corresponds to 250μm.

**Using linear regression to calculate No. of Virions binding / internalizaing**

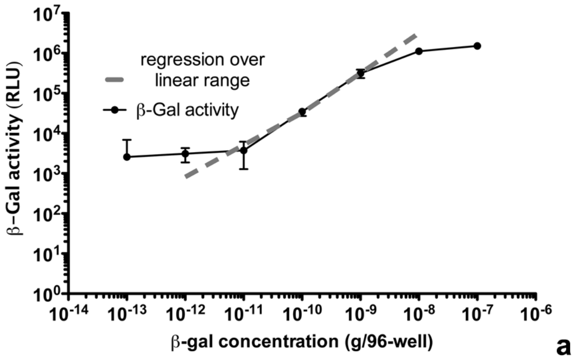


a

Calculating back from grams / well to molecules / well

Mw β−Galactosidase = 116519.7 (Da)
$N_A$ = 6.022 E23 (1/mol)
Mw/$N_A$ = 1.93 E-19 (g) per β−Galactosidase molecule

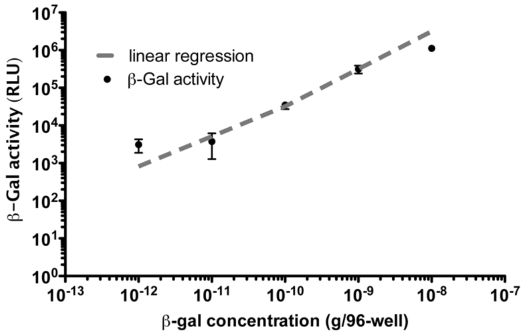


b

Regression over the linear range of the standard curve

y [RLU] = 6.014E-5*x [β−Gal/well] + 505.3

x = (y-505.3)/6.014E-5

**Comparing measurement results with estimated virion numbers**

Calculating the No. of active enzymes / well from RLU measurements

- Using the linear regression from the standard curve

Calculation No. of N proteins / well

No. of cells/24-well = 4.0E5

No. of N-proteins / MHV virion = 730-2200 [Ref. 31-33]
Assumption for calculations
No. N/virion = 1000

Calc. No. N/well = No. cells * MOI * No.N/virion
Calc. MOI = Calc No. act. enzymes / No. cells / (No. N/virion)

**BINDING**

| MOI | β-Gal activity (RLU) | Calc. No. of active enzymes / well | Calc. No. of N proteins / well | Calc. MOI |
|---|---|---|---|---|
| 1 | 30748 | 5.03E+08 | 4.00E+08 | 1.26 |
| 3 | 76870 | 1.27E+09 | 1.20E+09 | 3.17 |
| 10 | 303005 | 5.03E+09 | 4.00E+09 | 12.57 |

c

**INTERNALIZATION**

| MOI | β-Gal activity (RLU) | Calc. No. of active enzymes / well | Calc. No. of N proteins / well | Calc. MOI |
|---|---|---|---|---|
| 10 | 33004 | 5.40E+08 | 4.00E+09 | 1.35 |
| 30 | 82510 | 1.36E+09 | 1.20E+10 | 3.41 |
| 100 | 243213 | 4.04E+09 | 4.00E+10 | 10.09 |

d

**Figure S9.** Calculating virus binding and internalization using a standard curve. (**a**) Correlation between β-galactosidase activity and amount of β-galactosidase protein with indicated linear range. Increasing amounts of purified wild-type *E. coli* β-galactosidase were added to cell lysates of LR7ΔM15 cells. Samples were probed with Beta-Glo® substrate and incubated for 40 min at RT in the dark before measuring the β-galactosidase activity using a luminescence read-out. (**b**) Linear range of the β-galactosidase activity of the standard curve. (**c**) Calculation of number of active enzymes per well from the standard curve and correlation thereof with the estimated number of N proteins present in the binding assay based on literature.[1-3] (**d**) Calculation of the number of active enzymes per well from the standard curve and correlation thereof with the estimated number N proteins per well in the internalization assay.

## REFERENCES


1. Beniac DR, Andonov A, Grudeski E, Booth TF (2006) Architecture of the SARS coronavirus prefusion spike. Nature structural & molecular biology 13: 751-752.
2. Escors D, Camafeita E, Ortego J, Laude H, Enjuanes L (2001) Organization of two transmissible gastroenteritis coronavirus membrane protein topologies within the virion and core. Journal of virology 75: 12228-12240.
3. Neuman BW, Kiss G, Kunding AH, Bhella D, Baksh MF, et al. (2011) A structural analysis of M protein in coronavirus assembly and morphology. Journal of structural biology 174: 11-22.
